# Efficacy and safety outcomes of robotic radical hysterectomy in Chinese older women with cervical cancer compared with laparoscopic radical hysterectomy

**DOI:** 10.1186/s12905-018-0544-x

**Published:** 2018-05-01

**Authors:** Cheng Luo, Mei Liu, Xiuli Li

**Affiliations:** 1 Department of Gynecology, Chinese People’s Liberation Army General Hospital and Hainan Branch, Haitang Bay, Sanya, China; 2 Department of Oncology, Chinese People’s Liberation Army General Hospital and Hainan Branch, Sanya, China

**Keywords:** Cervical cancer, Chinese older women, Laparoscopic radical hysterectomy, Robotic radical hysterectomy

## Abstract

**Background:**

Recently, as a complex integrating a number of modern high-tech means, robotic surgery system is a well-deserved revolutionary tool in globally minimally invasive surgical field. For the first time in China, the objective of this study was to evaluate the efficacy and safety outcomes of robotic radical hysterectomy (RRH) in Chinese older women with cervical cancer compared with laparoscopic radical hysterectomy (LRH).

**Methods:**

In this prospective, randomized and double-blinded study, 60 Chinese older women with cervical cancer were evenly divided to accept the RRH or LRH. Follow-up period lasted for 24 months.

**Results:**

Median age for the entire cohort was 65 (range: 61-69) years. There was no difference in International Federation of Gynecology and Obstetrics (FIGO) stages and cell types between two groups (*p* > 0.05 for all). Uterine size, tumor size, vaginal length and numbers of left and right pelvic lymph nodes did not differ between two groups (*p* > 0.05 for all). No difference was observed in numbers of left and right lymph node metastasis (*p* > 0.05 for all). All patients had negative margins without conversion to laparotomy. There were significantly less postoperative complications in the RRH group than in the LRH group (*p* < 0.05). Shorter indwelling time of bladder and drain catheters was observed in the RRH group than in the LRH group (*p* < 0.05 for all). Length of postoperative hospital stay in the RRH group was significantly shorter compared with that in the LRH group (*p* < 0.05). Patients in two groups similarly experienced the recurrence and death (*p* > 0.05 for all).

**Conclusions:**

This study demonstrated that RRH provided additional benefits for Chinese older women with cervical cancer because of less complications and faster recovery compared with LRH. Meanwhile, this study supported an equivalence of surgical qualities and survival outcomes of RRH to LRH. Robotics-assisted surgical method is effective, safe and feasible for Chinese older women with cervical cancer.

## Background

Recently, as a complex integrating a number of modern high-tech means, robotic surgery system is a well-deserved revolutionary tool in globally minimally invasive surgical field [1]. It consists of three parts including high-resolution imaging device, surgeon operating platform and robotic manipulating arm, and adopts the most advanced master-slave remote control operation mode. The superiority provided by this new technology includes 3-dimensional magnified field, tremor filtration and wide range of instrument mobility, thus obviously addressing the ergonomic defects of traditional laparoscopic method [2, 3]. With increased precision and decreased wound, robotic surgery system has the opportunity to improve the efficacy and safety outcomes of surgical operation. Its clinical application has been developing rapidly and widely in various surgical fields.

Based on Cancer Statistics in China, there were 98,900 (expected incidence) new cases of cervical cancer in 2015, including 23,500 in Chinese older women (over the age of 60). Open radical hysterectomy (ORH) has been a main therapy of cervical cancer in China for a long time [4]. However, traditional surgical method results in uncomfortable symptoms and poor prognosis of patients due to a series of wounds and complications. Laparoscopic radical hysterectomy (LRH) has been confirmed to have the superiority over ORH through making the surgical operation more effective and safe [5, 6]. Several studies have also found the superiority of robotic assistance in solving the obstacles of ORH [7–10]. However, robotic radical hysterectomy (RRH) is an innovative technology with limited application to cervical cancer, and the studies analyzing its application in patients with cervical cancer are still insufficient all over the world [11–13]. Moreover, prospective studies comparing the efficacy and safety outcomes of RRH and LRH are scarce for older women [14, 15]. Meanwhile, robotic surgery system has just been applied in Chinese hospitals recently, and there are hardly any study assessing the clinical value of RRH on Chinese older women with cervical cancer. For the first time in China, the objective of this study was to evaluate the efficacy and safety outcomes of RRH in Chinese older women with cervical cancer compared with LRH.

## Methods

### Study patients and procedures

This study was a prospective, randomized and double-blinded analysis of Chinese patients older than 60 years with cervical cancer in gynecology department of our hospital conducted from January 2014 to January 2015. There were 60 patients evenly divided to accept the RRH or LRH using random numbers in a randomized block design. Random numbers were generated by Statistic Package for Social Science (SPSS) version 17.0 software (SPSS Inc., Chicago, USA). They all were diagnosed with cervical cancer at International Federation of Gynecology and Obstetrics (FIGO) stage no more than IIB, and had no severe abdominal adhesion and heart, lung, liver and kidney disorders. Patient characteristics and surgical details were recorded by well-trained investigators. Pathological specimens were read by experienced pathologists. Follow-up period lasted for 24 months, and no patient lost from the follow-up. Numbers of recurrence and death were the primary end-points. Total number of postoperative complications and length of postoperative hospital stay were the secondary end-points.

### Surgical techniques

All surgeries were conducted by gynecologists with experience in advanced laparoscopic procedures and made up of the following steps: 1) resection of round ligament of uterus; 2) pelvic lymphadenectomy; 3) ligation and dissection of uterine artery; 4) dissection of ureter; 5) resection of cardinal ligament of uterus and uterosacral ligament; 6) resection of upper vagina; and 7) vaginal cuff closure. RRH was conducted with a da Vinci system (Intuitive Surgical Inc., Sunnyvale, CA, USA). Five trocars were applied as follows: one 12-mm camera trocar placed via the open-Hasson technique, two 8-mm ancillary robotic trocars placed bilaterally (approximately 8-10 cm away from the camera port and 30 degrees below it to avoid the collisions between the robotic arms), and two assistant trocars. LRH was conducted as laparoscopically assisted operation with four trocars including one 12-mm camera trocar, two operating trocars and one assistant trocar. Nerve sparing technique was not conducted in all patients. After surgical operation, all patients underwent the paclitaxel plus cisplatin chemotherapy (TP regimen) and pelvic external radiotherapy.

### Statistical analyses

Statistical analyses were conducted using SPSS version 17.0 software (SPSS Inc., Chicago, IL, USA). Continuous variables with normal or skewed distribution were shown as mean (standard deviation) or median (interquartile range), and compared by Student’s t-test or Mann-Whitney U test, respectively. Categorical variables were shown as number (percentage), and compared by Chi-square test. Two-tailed *p*-values less than 0.05 were regarded as statistically significant.

## Results

Median age for the entire cohort was 65 (range: 61-69) years. Patient characteristics and pathological findings are shown in Table 1. Two surgical groups were similar with respect to age and history of previous pelvic surgeries (*p* > 0.05 for all). There was no difference in FIGO stages and cell types between two surgical groups (*p* > 0.05 for all). Uterine size, tumor size, vaginal length and numbers of left and right pelvic lymph nodes did not differ between two surgical groups (*p* > 0.05 for all). Numbers of left and right lymph node metastasis had no difference between two surgical groups (*p* > 0.05 for all). All patients belong to two surgical groups had negative margins.

**Table 1:** Patient characteristics and pathological findings

Characteristics	RRH group (*n* = 30)	LRH group (*n* = 30)	*P* value
Age^a^, yr	65(62-67)	64(62-66)	0.404
History of previous abdominal surgeries, *n* (%)	12(40.0)	15(50.0)	0.436
FIGO stages, *n* (%)			0.532
IA	5(16.7)	7(23.3)	
IB1	16(53.3)	14(46.7)	
IB2	1(3.3)	3(10.0)	
IIA	4(13.3)	4(13.3)	
IIB	4(13.3)	2(6.7)	
Cell types, *n* (%)			0.380
Squamous	18(60.0)	21(70.0)	
Adenoid	10(33.3)	8(26.7)	
Neuroendocrine	2(6.7)	1(3.3)	
Uterine size^a^, cm^3^	153.13(120.00-240.75)	191.29(147.09-223.62)	0.352
Tumor size^a^, cm^3^	4.95(1.43-9.54)	4.76(2.32-11.9)	0.767
Vaginal length^a^, cm	1.50(1.00-2.30)	1.35(1.00-2.00)	0.337
Left pelvic lymph nodes^a^, *n*	9.0(7.0-14.0)	8.5(6.0-13.0)	0.727
Right pelvic lymph nodes^a^, *n*	10.5(7.0-12.0)	10.0(7.0-11.0)	0.541
Left pelvic lymph node metastasis^a^, *n*	0(0-0)	0(0-1.0)	0.549
Right pelvic lymph node metastasis^a^, *n*	0(0-0)	0(0-0.5)	0.869

Notes: ^a^median (interquartile range). Abbreviations: *RRH* robotic radical hysterectomy, *LRH* laparoscopic radical hysterectomy, *FIGO* International Federation of Gynecology and Obstetrics

All patients were completed through RRH or LRH without conversion to laparotomy. Postoperative complications and survival outcomes are shown in Table 2. There were significantly less total number of postoperative complications in the RRH group than in the LRH group (*p* < 0.05). No significant difference between two surgical groups was seen in postoperative complications including febrile morbidity, port site cellulitis/hernia/dehiscence, urinary tract infection and ureteral injury (*p* > 0.05 for all). Two surgical groups had no postoperative complication including urinary retention, bowel injury/obstruction, pulmonary embolism and deep vein thrombosis. There was no postoperative complication of vaginal vault, such as bleeding, infection, dehiscence and leakage in two surgical groups. No postoperative complication including rectovaginal, vesicovagina and ureterovagina fistulas was noted in two surgical groups. Shorter indwelling time of bladder and drain catheters was observed in the RRH group than in the LRH group (*p* < 0.05 for all). Length of postoperative hospital stay in the RRH group was significantly shorter compared with that in the LRH group (*p* < 0.05). Patients in two surgical groups similarly experienced the recurrence and death. (*p* > 0.05 for all).

**Table 2:** Postoperative complications and survival outcomes

Characteristics	RRH group	LRH group	*P* value
Total number of Postoperative complications, *n* (%)	4(6.7)	11(18.3)	0.037
Febrile morbidity, *n* (%)	2(6.7)	5(16.7)	0.421
Port site cellulitis/hernia/dehiscence, *n* (%)	1(3.3)	3(10.0)	0.605
Urinary tract infection, *n* (%)	1(3.3)	2(6.7)	1.000
Ureteral injury, *n* (%)	0(0)	1(3.3)	1.000
Indwelling bladder catheter time^a^, d	6(5-11)	7(6-11)	0.043
Indwelling drain catheter time^a^, d	29(23-36)	32(28-38)	0.038
Length of postoperative hospital stay^a^, d	13(10-15)	15(11-17)	0.042
Recurrence, *n* (%)	2(6.7)	3(10.0)	1.000
Death, *n* (%)	1(3.3)	2(6.7)	1.000

Notes: ^a^median (interquartile range). Abbreviations: *RRH* robotic radical hysterectomy, *LRH* laparoscopic radical hysterectomy

## Discussion

Prevalence of cervical cancer has increased continuously over the last few decades in the entire globe, and ORH has been the most common surgical method in therapy of cervical cancer [4]. Subsequently, LRH has become the standard therapy of cervical cancer through providing the benefits of minimally invasive surgery [5, 6]. Recently, RRH has revolutionized the therapy of cervical cancer, and its application has been in development. Compared with ORH, previous studies have found that RRH leads to significantly fewer complications and shorter length of hospital stay [7–10]. While LRH is widely accepted for the therapy of cervical cancer, the numbers of patients with RRH have not shown the same growth partly due to a lack of studies supporting a better role of RRH than LRH in therapy of cervical cancer [11–13]. There exists a need for prospective studies to provide the evidence whether the robot provides additional benefits for Chinese older women with cervical cancer [14, 15].

For the first time in China, this study directly compared RRH with LRH in Chinese older women with cervical cancer. In addition to shorter indwelling time of bladder and drain catheters, patients accepting the RRH had significantly fewer postoperative complications and shorter length of postoperative hospital stay compared with LRH in this study, suggesting a faster recovery benefiting from RRH rather than LRH. In these aspects, RRH is more favorable to the therapy of cervical cancer compared with LRH because robotic instrument provides superior safety by means of its three-dimensional viewing, comfortable fatigue-reducing console and improved motion freedom, which significantly reduces the ergonomic problems of conventional laparoscopic instrument [2, 3].

Surgical qualities and survival outcomes are significant aspects to observe when applying a new surgical method. A 4-center study with 23 patients by Tinelli R has compared survival outcomes of robotic method to that of laparoscopic method on radical hysterectomy, and realized no difference in numbers of recurrence between two groups during the follow-up [16]. However, numbers of death are not evaluated in above-mentioned study. There was no significant difference between RRH and LRH with respect to numbers of recurrence and death in this study. In regard to surgical qualities, there were not only no conversion to laparotomy, but also no severe complication happened in patients accepting the RRH. Compared with LRH, surgical specimens removed during the RRH had not only similar uterine size, tumor size, vaginal length and numbers of pelvic lymph nodes (metastasis), but also negative margins, supporting the efficacy and feasibility of robotic method to radical hysterectomy.

This study had some limitations. Firstly, this study focused on Chinese older women. It is therefore difficult to generalize the findings to women with cervical cancer of all ages. However, prospective studies comparing the efficacy and safety outcomes of RRH and LRH are scarce for older Chinese women. Secondly, this study applied an approach based on country-specific recommendations and therefore the findings cannot be extrapolated to women with cervical cancer worldwide. However, we feel that the findings are relevant to the clinical practice in China.

## Conclusions

This study demonstrated that RRH provided additional benefits for Chinese older women with cervical cancer because of less complications and faster recovery when compared with LRH. Meanwhile, this study supported an equivalence of surgical qualities and survival outcomes of RRH to LRH. Robotics-assisted surgical method is effective, safe and feasible for Chinese older women with cervical cancer.

## Abbreviations

FIGO: International federation of gynecology and obstetrics
LRH: Laparoscopic radical hysterectomy
ORH: Open radical hysterectomy
RRH: Robotic radical hysterectomy
SPSS: Statistic package for social science
